# Green Synthesized Chitosan Nanoparticles for Controlling Multidrug-Resistant *mecA-* and *blaZ*-Positive *Staphylococcus aureus* and *aadA1*-Positive *Escherichia coli*

**DOI:** 10.3390/ijms25094746

**Published:** 2024-04-26

**Authors:** Aminur Rahman, Md Abdul Kafi, Geunyoung Beak, Sanjay Kumar Saha, Kumar Jyotirmoy Roy, Ahsan Habib, Tania Faruqe, Mahbubul Pratik Siddique, Md. Shafiqul Islam, Khandker Saadat Hossain, Jeong-Woo Choi

**Affiliations:** 1 Department of Microbiology and Hygiene, Bangladesh Agricultural University, Mymensingh 2202, Bangladesh; aminur50651@bau.edu.bd (A.R.); drsanjaysaha75@gmail.com (S.K.S.); jkroy39833@bau.edu.bd (K.J.R.); ahsan.50652@bau.edu.bd (A.H.); mpsiddique@bau.edu.bd (M.P.S.); shafiqdvm@yahoo.com (M.S.I.); 2 Department of Chemical and Biomolecular Engineering, Sogang University, Seoul 04107, Republic of Korea; beakgy@sogang.ac.kr; 3 Experimental Physics Division, Atomic Energy Centre, Dhaka 1000, Bangladesh; mimitaniabd@yahoo.com; 4 Department of Physics, University of Dhaka, Dhaka 1000, Bangladesh; k.s.hossain@du.edu.bd

**Keywords:** chitosan nanoparticles, green synthesis, MDR bacteria, antimicrobial, MIC, MBC, biocompatibility

## Abstract

Antimicrobial resistance has recently been considered an emerging catastrophe globally. The public health and environmental threats were aggravated by the injudicious use of antibiotics in animal farming, aquaculture, and croup fields, etc. Consequently, failure of antibiotic therapies is common because of the emergence of multidrug-resistant (MDR) bacteria in the environment. Thus, the reduction in antibiotic spillage in the environment could be an important step for overcoming this situation. Bear in mind, this research was focused on the green synthesis of chitosan nanoparticles (ChiNPs) using *Citrus lemon* (Assam lemon) extract as a cross-linker and application in controlling MDR bacteria to reduce the antibiotic spillage in that sector. For evaluating antibacterial activity, *Staphylococcus aureus* and *Escherichia coli* were isolated from environmental specimens, and their multidrug-resistant pattern were identified both phenotypically by disk diffusion and genotypically by detecting methicillin- (*mecA*), penicillin- (*blaZ*), and streptomycin (*aadA1*)-resistance encoding genes. The inhibitory zone’s diameter was employed as a parameter for determining the antibacterial effect against MDR bacteria revealing 30 ± 0.4 mm, 34 ± 0.2 mm, and 36 ± 0.8 mm zones of inhibition against methicillin- (*mecA*) and penicillin (*blaZ*)-resistant *S. aureus*, and streptomycin (*aadA1*)-resistant *E. coli*, respectively. The minimum inhibitory concentration at 0.31 mg/mL and minimum bactericidal concentration at 0.62 mg/mL of yielded ChiNPs were used as the broad-spectrum application against MDR bacteria. Finally, the biocompatibility of ChiNPs was confirmed by showing a negligible decrease in BHK-21 cell viability at doses less than 2 MIC, suggesting their potential for future application in antibiotic-free farming practices.

## 1. Introduction

Chitosan has numerous biomedical applications due to its remarkable antibacterial activity, biocompatibility, biodegradability, and self-renewal [1]. With its potential as an effective alternative to commercial antibiotics, it may help curb antimicrobial resistance (AMR). Here, we synthesized chitosan nanoparticles (ChiNPs) using an eco-friendly method with the aim of controlling environmental multidrug-resistant (MDR) bacteria. AMR, caused by residual antimicrobial agents in the food chain and the environment, poses a serious threat to public health [2,3,4,5]. This risk is exacerbated by increased agricultural activity, including aquaculture and livestock husbandry, to meet the rising demand for food, mainly due to rapid population growth in developing countries. To meet the high food demand, farmers widely use antibiotics without strict adherence to antibiotic withdrawal periods [6]. Consequently, a significant proportion of residual antibiotics can be found as a residue in food channels or as spillover in the environment, leading to significant public health challenges. Numerous studies in shrimp farms have detected *Staphylococcus aureus* (*S. aureus*) and *Escherichia coli* (*E. coli*) resistant to various antibiotics, including trimethoprim, azithromycin, levofloxacin, gentamicin, ampicillin, polymyxin, co-trimoxazole, ciprofloxacin, oxytetracycline, neomycin, and cephalexin [7]. In addition, various antibiotic-resistance genes, including methicillin-resistance genes (*mecA*, *mecB*, and *mecC*), a penicillin-resistance gene (*blaZ*), and others (*vanB*, *tetA*, *tetB*, and *tetC*), have been reported. *S. aureus* with enterotoxin-producing toxic shock syndrome toxin 1 and *E. coli* carrying resistance genes for tetracycline, ampicillin, trimethoprim, sulfamethoxazole-trimethoprim, and streptomycin (*tetA*, *tetB*, *tetC*, *tetD*, *tetE*, *tetG*, *stx1*, *aadA1*) have been detected on shrimp farms and hatcheries [7,8,9,10]. Plasmids containing resistance genes can be transmitted horizontally to other microbes in the environment [11]. Consequently, medically important bacteria can acquire resistance to commonly used antibiotics [12]. Antibiotic-resistance genes are further spread through the distribution of antimicrobial residue-containing food through farm-to-fork channels [13]. Therefore, the introduction of novel antimicrobials as alternatives to agricultural antibiotics, especially in shrimp farms, may be effective in reducing antimicrobial residues in the food chain. A recent review highlighted the application of antimicrobial nanomaterials to tackle this challenge [14]. Gao et al. (2021) proposed that ongoing research and development can lead to nanomaterials emerging as a primary approach in combating bacterial infections in the face of increasing antibiotic resistance [14]. This study sought to develop and assess the use of antimicrobial nanomaterials as an alternative to antibiotics to curb antibiotic spillover into the environment. Numerous natural antimicrobial materials have been previously used for this purpose [15,16,17]. However, their application has been limited by high costs, labor intensiveness, and the bio-incompatibility of their extraction and nanoformulation processes [18,19,20]. Therefore, cost-effective, simple, and eco-friendly methods of synthesizing antibacterial nanomaterials are critically needed.

The use of nanomaterials as alternatives to antibiotics has drawn significant attention due to their enhanced functionality, long-acting effects, surface-to-volume ratio, chemical complexation properties, high surface areas, enhanced ion-exchange capacity, low toxicity, high stability [21], and numerous potential applications in science and technology [21,22,23,24]. Metal, metal oxide, organic, and hybrid nanoparticles (NPs), such as AgNPs, ZnNPs, and TiO_2_NPs, have been utilized in versatile biomedical [25,26,27] and industrial applications [28,29]. However, they are not feasible for therapeutic applications due to their lack of biocompatibility [30]. Thus, efforts have been made to develop eco-friendly methods for synthesizing biocompatible nanoparticles. The recently promoted biological approaches like plant extracts as well as microbe-based synthesis of nanomaterials have filled the void of bio-incompatibility, exhibiting superiority over the chemical approaches [25,31,32]. It has been reported that biological/green synthesized nanomaterials have been effectively controlling many endemic diseases with less adverse effects [33]. Thus, the trend of using natural products as material sources has increased, and the active plant extracts and microbial agents are frequently employed for new drug discovery [25,29,34,35,36]. Chitosan oligosaccharide, which can be extracted from chitinous organisms, has gained attention for its abundance in nature, safety, biodegradability, self-renewability, and antimicrobial activity [37]. Chitosan has many applications in pharmaceuticals, drug delivery, biotechnology, cosmetics, textiles, and wastewater treatment [23,38,39,40,41,42]. In vitro studies indicate that ChiNPs exhibit a broad spectrum of antibacterial activity [43], as well as modest effects against yeast, mold, and viruses [44]. As a drug carrier, chitosan nanoparticles facilitate the transport of insulin, rifampicin, estradiol, etc., to penetrate the targeted sites by paracellular and transcellular transportation [45]. In agriculture, chitosan nanoparticles have been used as nano fertilizers resulting in the slow release of fertilizer [46]. The increased surface areas and increased electrostatic properties with amino and hydroxyl groups of chitosan nanoparticles facilitated the removal of heavy and dry metals from waste materials [47]. During the last decades, chitosan nanostructures became popularized in the field of biosensor fabrication for the application of medical and veterinary diagnostic tools, and in agriculture, the food industry, tissue engineering, and environmental monitoring [1,37,48,49,50]. Recently chitosan nanoparticles have been employed as nano adjuvants in animal and human vaccine developments [51,52]. However, they have limited biocompatibility and suitability for in vivo use because the synthesis process involves various chemicals, including sodium tripolyphosphate, sodium borohydride, sodium bis-(2-ethylhexyl) sulphosuccinate, acetone, methanol, and glutaraldehyde as reducing/capping agents [53] (Table 1). Therefore, we hypothesized that an eco-friendly method of synthesizing ChiNPs using a natural extract as a crosslinker can avoid the use of chemical ingredients, overcoming bio-incompatibility. The identification of a natural reducing agent/crosslinker to replace chemical reduction is crucial in addressing bio-incompatibility [54]. Moreover, determining the cost-effectiveness and eco-friendliness of methods that avoid chemicals in extracting, purifying, and synthesizing nanoparticles is an important research topic in bio-nanoengineering [20]. Nanotech products generated using green methods would be bioresorbable, biodegradable, and self-renewable in biological and ecological systems, reducing the burden on the environment.

In this study, we used lemon extract as a crosslinker and shrimp chitin as a source of chitosan oligosaccharide in an eco-friendly protocol for synthesizing ChiNPs. The resulting product was physically characterized using a UV–vis spectrophotometer (UV–vis), dynamic light scattering (DLS), X-ray diffractometry (XRD), scanning electron microscopy (SEM), atomic force microscopy (AFM), anfid transmission electron microscopy (TEM). Chemical reactions occurring during the particle-generation process were confirmed through a Fourier transform infrared spectroscopic (FTIR) analysis. The antibacterial activity of the physically characterized ChiNPs was evaluated against methicillin-resistant (*mecA*) and penicillin-resistant (*blaZ*) *S. aureus*, as well as streptomycin-resistant (*aadA1*) *E. coli* isolated from a shrimp farm. The minimum inhibitory concentration and minimum bactericidal concentration (MBC) of the ChiNPs were evaluated against the isolated MDR bacteria. The biosafety of the ChiNPs was assessed using BHK-21 cells.

## 2. Results

### 2.1. Green Synthesis of Chitosan Nanoparticles (ChiNPs)

**Figure 1:**
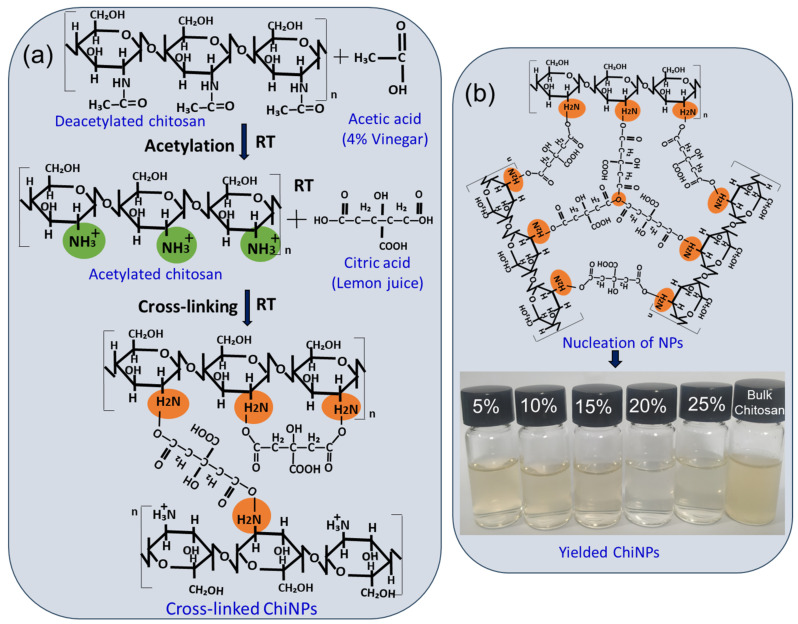
Schematic illustration of the chemistry involved in the formation of chitosan nanoparticles. (**a**) Acetylation of deacetylated chitosan followed by cross-linking with citric acid for enucleation of particle formation. (**b**) Growth of chitosan nanoparticles with varying concentrations (5–25%) of lemon extract.

ChiNPs were synthesized from chitosan powder using freshly extracted lemon juice (citric acid) as the crosslinker. The formation of ChiNPs was initiated by dissolving deacetylated chitosan in DIW through acetylation with vinegar (acetic acid), leading to the formation of the NH_3_^+^ group by removing H_3_C-C=O from deacetylated chitosan to acetylated chitosan (Figure 1). Crosslinking was then achieved through the bonding of the NH_3_^+^ groups to the OH group of the cross-linker (lemon citric acid [C_6_H_8_O_7_]) [67] to form a CO–H_2_N bond between acetylated chitosan and the crosslinker to form ChiNPs. Continued stirring allowed all the NH_3_^+^ groups to crosslink with the OH groups of the crosslinker. Thus, ChiNP nucleation occurred first, followed by the growth reaction over time to form stable nanoparticles (Figure 1a,b).

**Figure 2:**
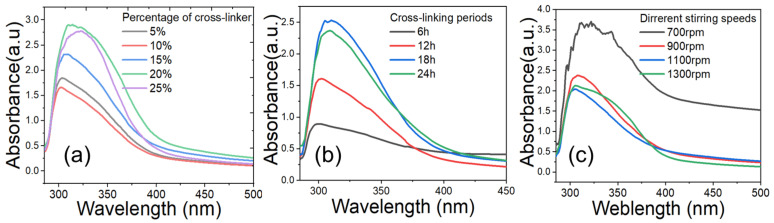
UV–vis spectra analysis of yielded ChiNPs. (**a**) Different concentration of cross-linker, (**b**) different cross-linking periods using 20% cross-linker, and (**c**) different stirring speeds using 20% cross-linker.

The synthesis process was initially monitored by observing visible changes in the transparency of the solution during the reduction process, which revealed that the maximum transparency of the ChiNPs solution was achieved when the crosslinker was used at 20%, while at 5%, 10%, 15%, and 25%, the crosslinker resulted in less transparency (Figure 2a, inset). The transparency of the ChiNPs solution results from the scaling down of bulk materials to nanoscale size, which allows maximum light passage [68]. Thus, the maximum transparency achieved with the crosslinker at 20% indicated complete crosslinking of bulk chitosan, resulting in the maximum ChiNP yield [69]. The yellowish, low transparency observed when using the crosslinker at 5%, 10%, 15%, and 25% indicated a lower ChiNP yield due to incomplete crosslinking.

### 2.2. UV–Vis Spectroscopic Analysis to Determine Particle Yield

The concentration of ChiNPs in the synthesis solution was determined using UV–vis analysis at a wavelength range of 300–320 nm, which corresponds to the characteristic absorption peak (λ_max_) of ChiNPs [53] (Figure 2). The highest λ_max_ peak of the product obtained with 20% lemon juice indicated that the transparency data aligned with the maximum ChiNP yield [53]. The relatively lower intensity of the λ_max_ peak at 300–305 nm in products from 5%, 10%, 15%, and 25% of the crosslinker indicated a lower production of ChiNPs with larger sizes due to suboptimal crosslinker proportions and particle sources (Figure 2a) [53]. Similarly, the maximum λ_max_ peak at 310 nm observed after 18 h of crosslinking indicated complete crosslinking, whereas the minimum λ_max_ peak at 300–325 nm after 6–24 h of reduction indicated incomplete crosslinking of the bulk chitosan (Figure 2b) [70]. Additionally, the maximum λ_max_ peak at 308 nm observed after stirring at 1100 rpm indicated complete chitosan crosslinking, whereas the minimum λ_max_ peak at 295–302 nm observed after stirring at 700–1300 rpm was because of incomplete crosslinking (Figure 2c) [71]. Overall, these data indicate that treating the chitosan solution with 20% lemon juice while stirring at 1100 rpm for 18 h resulted in the highest concentration of ChiNPs. These conditions were therefore selected to achieve maximum ChiNP production.

### 2.3. NP Analyses Using DLS and XRD

**Figure 3:**
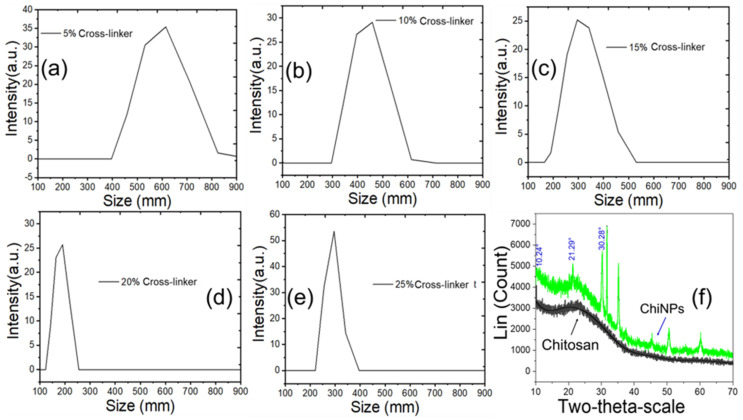
Dynamic light scattering (DLS) of yielded ChiNPs from different concentrations of cross-linker (**a**) 5%, (**b**) 10%, (**c**) 15%, (**d**) 20%, (**e**) 25%, and (**f**) XRD of chitosan and chitosan nanoparticle.

The hydrodynamic size distribution of the synthesized ChiNPs was determined using DLS analysis (Figure 3) [72]. This analysis revealed that the particle size ranged from 150 nm to 620 nm when different concentrations of lemon juice were used. ChiNPs produced using 20% lemon juice had an average nanoscale size of 150–250 nm (Figure 3d), consistent with complete chitosan crosslinking as indicated by the UV–vis data [73]. However, using lemon juice at 5%, 10%, 15%, and 25% resulted in ChiNPs with sizes of 400–620, 300–600, 230–530, and 220–400 nm, respectively (Figure 3a–c,e), indicating incomplete particle synthesis [73]. The crystallinity of the yielded ChiNPs was determined by means of X-ray diffraction (XRD) analysis. XRD analysis of the ChiNPs demonstrated sharp diffraction with high-intensity peaks at 10.24°, 15.31°, 21.29°, 30.29°, 31.7°, 35.28°, 37.52°, 45.44°, 50.51°, and 60.21° (Figure 3f) and a low full-width half maximum of 0.421° which indicated that the particles were crystal in nature. The average crystallite size of ChiNPs obtained from the Debye–Scherrer formula was 17 nm. The observed diffraction reflection was well matched with previous studies indicating that the three distinctive peaks 2θ at 10.24°, 21.29°, and 30.28°, which were the observed result from ChiNP formation [74,75,76]. The decrease in intensity of the XRD peaks at approximately 2θ = 15.31° and 21.29° indicates crosslinking between chitosan and citric acid, as previously reported [77,78]. Earlier studies indicate that the other strong peaks at 2θ of 31.7°, 35.28°, 37.52°, 45.44°, 50.51°, and 60.21° revealed by XRD analysis are associated with the crystalline structure of the ChiNPs [79,80,81].

### 2.4. Step-by-Step Confirmation of ChiNPs Synthesis Using FTIR

**Figure 4:**
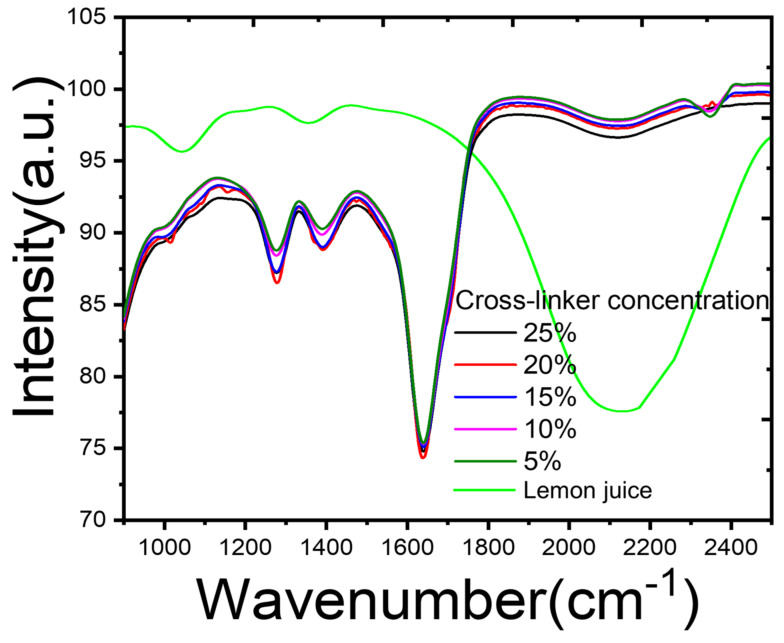
FTIR investigation of synthesized ChiNPs to confirm the functional groups formed during the cross-linking process.

The infrared spectra of various functional groups formed during ChiNP synthesis were determined using FTIR analysis in the range of 900–2500 cm^−1^ (Figure 4). The FTIR spectra of the ChiNPs from the 20% crosslinker (indicated in red) exhibited two distinct intense bands at 1015 cm^−1^ and 1157 cm^−1^, indicating the presence of C–O–C and H_3_N^+^, respectively, thereby confirming chitosan short oligomer crosslinking during synthesis. The appearance of the C–O, the secondary amine (–NH), the amino (–NH_2_), and the O–H groups indicated chitosan acetylation [82]. Deacetylated chitosan was aggregated by the citric acid crosslinker (in the lemon juice), thereby forming H_3_N^+^ stretching at 1157 cm^−1^ [83]. This zwitterion may be formed because of the solution’s pH change during lemon juice-mediated crosslinking of adjacent chitosan oligomers, where CA forms a covalent link with H_3_N^+^ groups at one end and with the C–O–C group at the end. This phenomenon was confirmed by the appearance of the two distinct stretching at 1157 cm^−1^ and 1015 cm^−1^, respectively, in the chitosan crosslinked using 20% lemon juice for ChiNP nucleation. This H_3_N^+^ abundance provides the positive charge behavior of the ChiNPs, which is critical for their antimicrobial effects [84]. Positively charged nanomaterials are thought to interact with negatively charged bacterial cell walls through electrostatic interaction [85]. This hypothesis was tested by evaluating the antibacterial effects of the ChiNPs against MDR bacteria isolated from shrimp farms. A weak band generated at 2308 cm^−1^ due to the presence of methylene C=H groups, which was not assigned for the 20% crosslinker, is due to the degradation of polysaccharides reduced for the formation of short oligomers, as shown in the red color line in Figure 4. Many common mild intense bands were generated at the 1280 cm^−1^, 1403 cm^−1^, 1678 cm^−1^, and 2130 cm^−1^ spectral regions due to the presence of C–O, the secondary amine of –NH, the amino group of –NH_2_, and –OH, which were observed in the green, pink, blue, and black spectra obtained with a 5%, 10%, 15%, and 25% crosslinker, as shown in Figure 4. The generation of such common spectra indicates the presence of chitosan.

In the case of the squeezed lemon solution, the high spectral regions at the 2000–2300 cm^−1^ bands generated by the O–H group and the reduced intensity of the ChiNP band in the same region indicated a decrease in OH groups through crosslinking with chitosan during nanoformulation. The formation of several weak intense bands at 778 cm^−1^, 1105 cm^−1^, and 1712 cm^−1^ was due to the presence of C=O, C–OH, and CH_2_. As previously reported, such spectra form because of the existence of citric acid in the solution [86,87].

### 2.5. Morphological Investigation with AFM

**Figure 5:**
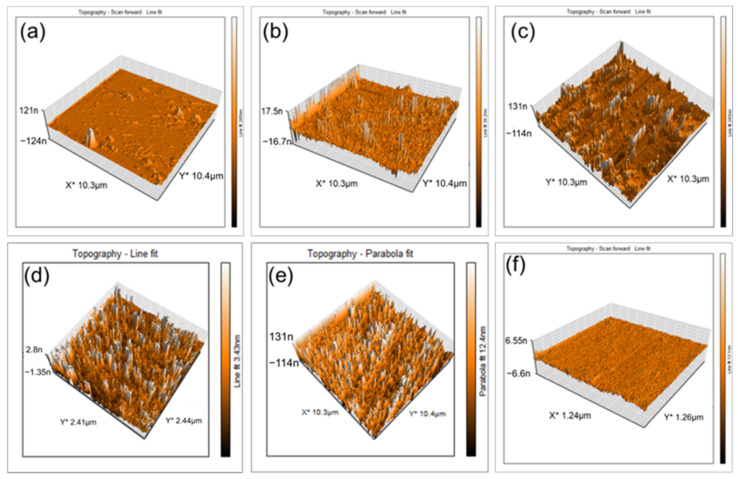
Topographic AFM images of yielded ChiNPs based on various concentrations of cross-linker: (**a**) 5%, (**b**) 10%, (**c**) 15%, (**d**) 20%, (**e**) 25%, and (**f**) control surface.

Three-dimensional topographic AFM image analysis was used to determine the morphology and dimensions of the ChiNPs produced using 20% lemon juice, which exhibited maximum homogenous nanoparticle distribution (Figure 5d) due to the presence of a sufficient amount of the crosslinker for complete chitosan crosslinking [66]. Relatively larger, non-uniformly distributed ChiNPs were obtained using 15% and 25% lemon juice (Figure 5c,e), whereas fewer ChiNPs were generated using 5% and 10% lemon juice (Figure 5a,b) because of insufficient amounts of the crosslinker [66]. No particles were observed on the control surface (Figure 5f).

### 2.6. Surface Topographic Analysis Using SEM

**Figure 6:**
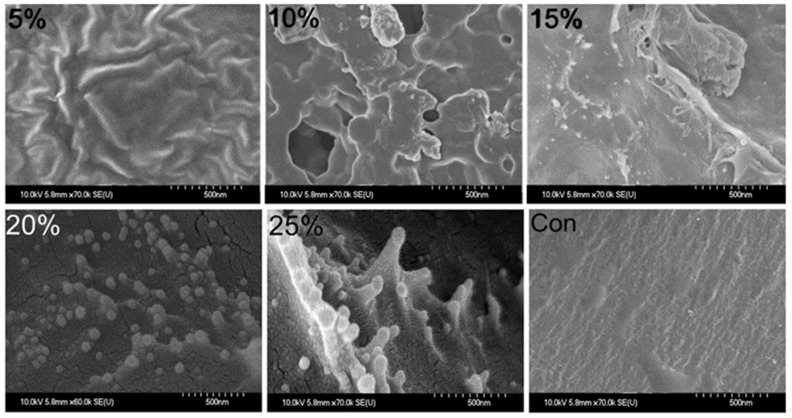
SEM images of yielded ChiNPs varying concentrations of cross-linking agent.

Topographic analysis of the SEM images revealed that using 20% lemon juice to crosslink polymeric chitosan resulted in the highest amount of homogenously dispersed spherical nanoparticles, yielding ChiNPs with a size of 150–250 nm. However, when 25% lemon juice was used as the crosslinker, relatively larger, irregularly shaped, non-homogenously dispersed ChiNPs were obtained. In contrast, 5%, 10%, and 15% lemon juice crosslinkers yielded very few nano-scale particles. No particles were observed on the control surface (Figure 6). Taken together, all physical characterization data indicate that 20% lemon juice is the optimal concentration for chitosan oligosaccharide crosslinking, as it leads to the highest ChiNP yield, consistent with previously reported hydrodynamic observations [88,89].

### 2.7. ChiNPs Analysis Using TEM

**Figure 7:**
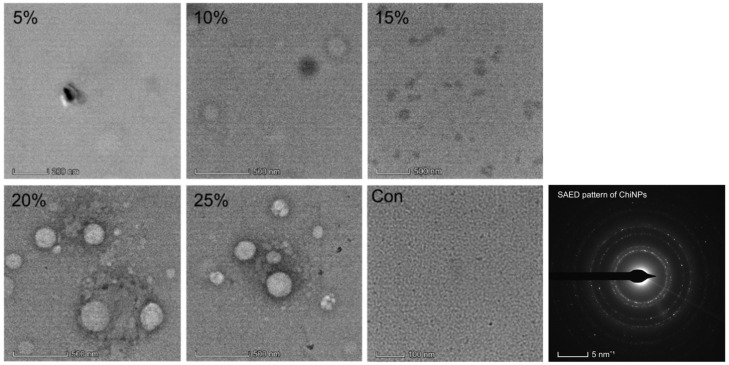
The TEM images of yielded ChiNPs with various concentrations of cross-linking agent.

Analysis of the TEM images revealed that the use of a 20% crosslinker resulted in the production of round-shaped ChiNPs with a size of 100–250 nm, whereas the use of 25% crosslinker led to the formation of relatively larger, aggregated ChiNPs. No particles were observed when 5%, 10%, or 15% crosslinker was used, as well as in the control images (Figure 7).

### 2.8. Identification and Characterization of MDR Bacteria

**Figure 8:**
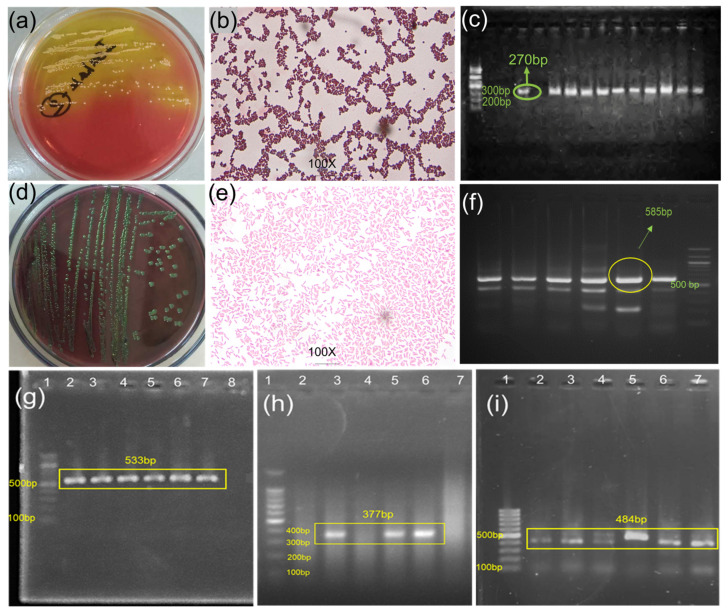
Isolation and identification of MDR bacteria: (**a**) Colony morphology and cultural characteristics, (**b**) staining properties, and (**c**) species-specific amplicon (270 bp) of gene (*nuc*) of *S. aureus*. (**d**) Colony morphology and cultural characteristics, (**e**) staining properties and (**f**) species-specific amplicon (585 bp) of *16srRNA* gene of *E. coli*. (**g**) amplification of methicillin-resistant *mecA* gene (533 bp), (**h**) penicillin-resistant *blaZ* gene (377 bp) for *S. aureus* and (**i**) streptomycin-resistant *aadA1* gene (484 bp) for *E. coli*. For every case, Lane1 was a 100 bp DNA ladder.

The bacteria were isolated and identified using culture, Gram staining, and PCR amplification of the *Staphylococcus*-specific genes *nuc* and *16S rRNA*. *Staphylococcus aureus* was identified by the presence of round, yellowish colonies with the appearance of yellowish color change of mannitol salt agar plates, by the appearance of purple-colored cocci, grape-like bunches under the microscope, and by the presence of a 270-bp *nuc* gene PCR product (Figure 8a–c) [90]. *E. coli* was identified by the presence of round-shaped, green colonies with a metallic sheen on eosin–methylene blue agar plates, by their appearance as single, short, rod-shaped pink-colored colonies under the microscope, and by the presence of a 585-bp *16S rRNA* gene PCR product (Figure 8d–f) [90]. After species identification, the bacteria were used for phenotypic and genetic assessment of multidrug resistance.

Antibiotic resistance was first assessed through antibiogram analysis of the activity of various classes of commercial antibiotics against PCR-confirmed bacterial species using disk diffusion methods [7,10]. This analysis revealed that 18 *S. aureus* isolates were resistant to at least 6 of the 11 antibiotics (6 antibiotic classes) (Figure S1a), whereas 19 *E. coli* isolates were resistant to at least 5 of the 11 antibiotics (5 antibiotic classes) (Figure S1b). Bacteria that exhibit resistance to three or more classes of antimicrobial drugs are classified as MDR [53,91]. Next, the PCR analysis was used to detect the presence of antibiotic resistance genes against methicillin (*mecA*, 533 bp) and penicillin (*blaZ*, 377 bp) in phenotypically confirmed MDR *S. aureus* isolates (Figure 8g,h), as reported previously [10,92]. Similarly, the presence of the streptomycin resistance gene *aadA1* (484 bp) was detected in *E. coli* (Figure 8i), as described in previous studies [7,10].

### 2.9. Determination of the Antibacterial Activity of ChiNP

**Figure 9:**
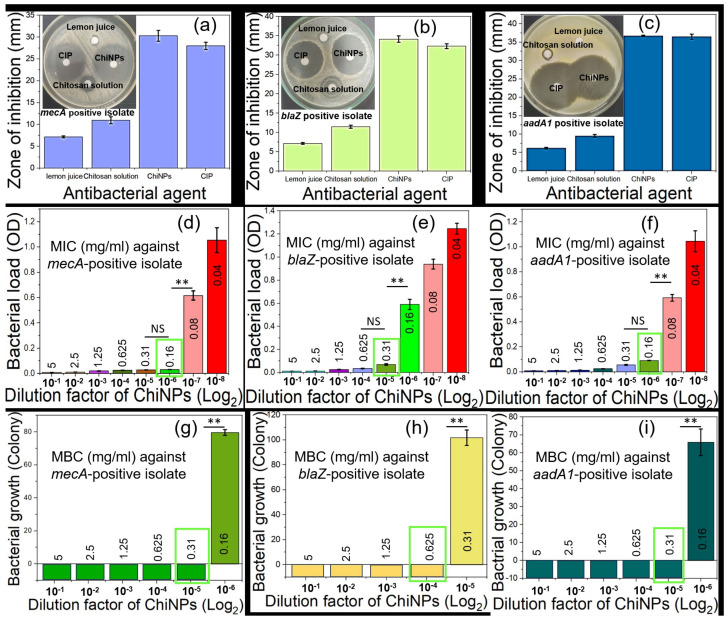
Antibacterial effect of yielded ChiNPs against *mecA* (**a**) and *blaZ* (**b**) positive *S. aureus*, and *aadA1* positive *E. coli* (**c**). Minimal inhibitory and bactericidal concentrations (MIC and MBC, respectively) of ChiNPs for *mecA-* (**d**,**g**) and *blaZ* (**e**,**h**)-positive *S. aureus*, and *aadA1*-positive *E. coli* (**f**,**i**). (NB: ** represents the very significant and Green boxes indicate MIC and MBC values at the highlighted dilution point).

The MDR-confirmed *S. aureus* and *E. coli* isolates were evaluated for their antibacterial effects by measuring zones of inhibition surrounding ChiNP (selected from antibiogram of ChiNPs yielded after employing different conditions; see Figure S2)-impregnated disk (1 mg/disk) as a parameter to determine sensitivity against bacteria. The antibiogram analysis revealed that the zones of inhibition against *S. aureus* resistant to methicillin (*mecA*) and penicillin (*blaZ*) were 30 ± 0.4 and 34 ± 0.2, respectively (Figure 9a,b). For streptomycin-resistant (*aadA1*) *E. coli*, the zone of inhibition was 36 ± 0.8 mm (Figure 9c). Moreover, bulk chitosan solution (used at the same dose as the iron source) resulted in significantly smaller zones of inhibition (10 ± 0.9 mm, 11 ± 0.5 mm, and 9 ± 0.8 mm), and lemon juice alone (at 20% concentration for reduction) did not produce any zones of inhibition. The zones of inhibition from ciprofloxacin, used as a positive control, did not differ significantly from ChiNPs, highlighting the effectiveness of ChiNPs against MDR bacteria. Moreover, the intermediate-sized zones of inhibition caused by bulk chitosan solution and the lack of lemon juice-associated zones of inhibition indicated that the ChiNP-associated zones of inhibition were due to their nanoformulation. Taken together, these observations indicate that the ChiNPs synthesized using our green method are also effective against antibiotic-resistant bacteria.

The MIC of the ChiNPs was determined using OD analysis of bacterial broth cultures treated with various doses of NPs for 24 h at 37 °C (Figure 9d–f). This analysis revealed that the MICs of the ChiNPs against 1.9 × 10^5^ CFU/mL (see Figure S3) of methicillin-resistant (*mecA*) and penicillin-resistant (*blaZ*) *S. aureus* were 0.16 mg/mL and 0.31 mg/mL, respectively (Figure 9d,e), whereas against streptomycin-resistant (*aadA1*) *E. coli*, the MIC was 0.16 mg/mL (Figure 9f). Likewise, the MBC of the ChiNPs was also determined by observing bacterial growth on selective media inoculated from 24 h incubated NP diluted broth. This analysis revealed that against methicillin-resistant (*mecA*) and penicillin-resistant (*blaZ*) *S. aureus*, the ChiNPs had MBC values of 0.31 mg/mL and 0.62 mg/mL, respectively (Figure 9g,h), and against streptomycin-resistant (*aadA1*) *E. coli*, the MBC value was 0.31 mg/mL (Figure 9i). These observations indicate that methicillin-resistant and penicillin-resistant *S. aureus*, as well as streptomycin-resistant *E. coli*, were highly sensitive to the ChiNPs. Moreover, the MIC and MBC values of 0.31 mg/mL and 0.62 mg/mL, respectively, are compatible against both Gram-positive and Gram-negative bacteria. Together, these findings indicate that in commercial farms, ChiNPs could serve as an effective alternative to antibiotics for producing antibiotic residue-free food and reducing AMR by minimizing antibiotic contamination in the environment.

### 2.10. Biosafety Evaluation of ChiNPs

**Figure 10:**
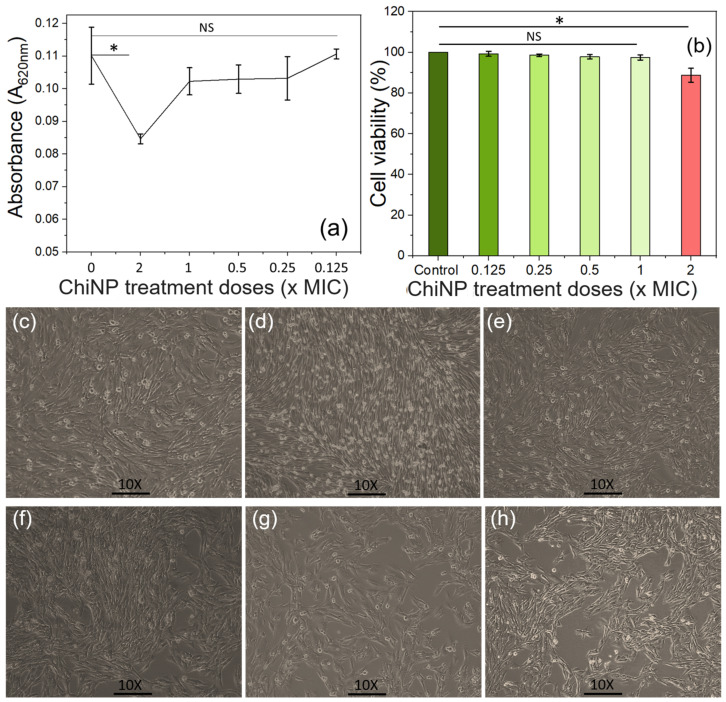
Effect of ChiNPs on the viability of BHK-21 cells. (**a**) OD of various doses (MICs) of ChiNP-treated cells, (**b**) cell viability assay of various doses (MICs) of ChiNP-treated cells, (**c**) non-non-treated control BHK-21 cells at 72 h post seeding, (**d**) 0.125 × MIC doses of ChiNP-treated cells, (**e**) 0.25 × MIC doses of ChiNP-treated cells, (**f**) 0.5 × MIC doses of ChiNP-treated cells, (**g**) 1 × MIC doses of ChiNP-treated cells and, (**h**) 2 × MIC doses of ChiNP-treated cells at 24 h post-treatment. (NB: * represents the very significant decrease of cell viability and NS represents the non-significant difference of cell viability).

For any therapeutic substance, biosafety compliance is crucial before approval for in vivo use. Here, the biosafety of ChiNPs was evaluated on the BHK−21 cell line (Figure 10c,d) using the MTT assay (see Figure S4) [93]. This assay is based on the principle that the mitochondrial reductase enzyme in viable cells reduces MTT into insoluble formazan dye, which, upon hydrolysis by DMSO, produces a colored solution [93]. The OD of this solution therefore reflects cell viability [94]. This analysis did not reveal a significant (0.01 ˂ *p* ≤ 0.05) difference in cell viability between the control cells and the cells treated with NPs at MIC doses (Figure 10a,b). A significant difference in cell viability was observed using double the MIC dose; however, approximately 90% of viable cells were still observed. Taken together, these observations indicate that the ChiNPs generated using our eco-friendly process are biocompatible for in vivo applications. In the near future, applying this greenly yielded ChiNP to antibiotic-free safe livestock farming could be explored to produce consumer-safe, healthy, and sustainable foods of animal origin. Though the yielded ChiNPs exhibit potential antibacterial effects against MDR bacteria, the antiviral as well as antifungal activity of the yielded NPs need to be verified before field application.

## 3. Materials and Methods

### 3.1. Materials

Low-molecular-weight chitosan powder and phosphate-buffered saline were purchased from Sigma–Aldrich, Waltham, MA, USA. Antibiotic disks, blotting paper, and bacterial culture media (Mueller–Hinton, Mannitol Salt, Nutrient Agar, Eosin-methylene Blue, and Nutrient Broth Agar) were purchased from Hi-Media Laboratories Pvt. Ltd., Mumbai, India. Vinegar (acetic acid) was purchased from Qualikems Fine Chem Pvt. Ltd., Dhaka, Bangladesh. Chemicals including alcohol and disinfectants were acquired from ZH Chemicals (Hatkhola, Dhaka, Bangladesh). Gram staining materials were obtained from the laboratory stock at the Microbiology and Hygiene Department. All the chemicals were obtained at a purified grade and used as received.

### 3.2. Preparation of Chitosan Solution and Lemon Extract

To obtain a 1% chitosan solution, 500 mg deacetylated chitosan powder was added to 44.6 mL of deionized water (DIW), and the mixture was stirred at 1200 rpm for 1 h at 37 °C until dissolved completely. Deacetylated chitosan was then acetylated by adding 4.4 mL of vinegar in a dropwise manner to obtain a thick jelly like solution. To prepare the lemon extract, fresh *Citrus lemon* (Assam lemon) was washed, chopped, and then squeezed using a hydraulic lemon squeezer. The resulting solution was centrifuged at 1300 rpm for five minutes to remove fibrous components and collect the supernatant.

### 3.3. Synthesis of Chitosan Nanoparticles (ChiNPs)

The purified lemon extract was mixed with a chitosan solution to prepare various concentrations (5% to 25%) and crosslinked for various periods (6 to 30 h) at various stirring speeds (700 to 1300 rpm) at room temperature for a maximum nanoparticle yield. After the solution’s color shifted from yellowish to transparent, the mixture was centrifuged at 1300 rpm for 10 min. The supernatant was collected and stored at 4 °C.

### 3.4. Physical Characterizations of the ChiNPs

The concentration of the ChiNPs was determined using a UV–vis spectrophotometer (UV-1600PC) at wavelengths ranging from 259 to 510 nm, using DIW as the reference. Data from both the blank chitosan solution and the ChiNP solution were compared by evaluating the shifts of the λ_max_ peak. Particle size distribution and average particle size were measured using DLS at a scattering angle of 90°. The crystallinity of the nanoparticles was determined through XRD analysis. For X-ray diffraction, the ChiNP solution was dried on a silicon wafer after drop-casting and then scanned at dispersion angles of 5° and 80° (Rigaku Smart Lab XRD: PW1730, I ^1/4^ 1.54056A [Cu K^0^ an irradiation] 2q ^1/4^ 20–70^0^). The morphological features of the spin-coated (at 1300 rpm and dried in an oven at 600 °C) ChiNPs were examined using AFM (Nanosurf Flex AFM) in contact mode with a cantilever to determine particle size and dimensions. The external morphology, crystalline structure, and particle size were assessed using SEM (FE-SEM, Hitachi S-4700). A thin gold (Au) layer was deposited over the NP-coated silicon wafer to achieve material conductivity, followed by imaging at an acceleration voltage of 10 kV with a current of 10 mA. TEM (Talos F200X G2) 4 k × 4 k images (pixel: 512 × 512) were acquired at an acceleration voltage of 80 kV using a Ceta 16 M camera at 320 fps. Finally, elemental analysis of the yielded NPs was conducted using FTIR in basic mode (Golden Gate) at a resolution of 4–64 cm^−1^ scan rates using PerkinElmer IR spectroscopy software version 10.4.3.

### 3.5. Isolation and Identification of MDR Bacteria

To isolate MDR bacteria, 30 samples of shrimp farm water (Figure S5) were collected from Satkhira, Bangladesh, and inoculated into nutrient broth for enrichment. The enriched inoculum was streaked onto plates with bacteria-specific media (e.g., eosin methylene blue and mannitol salt agar) and incubated at 37 °C for 24 h to obtain single colonies. The single colonies recovered from the selective media plates were subjected to Gram staining, as described before [95]. Species-specific amplification of the *nuc* and *16S rRNA* genes was used to confirm the identity of *S. aureus* and *E. coli* colonies (Table 2), which were then tested for the presence of MDR genes. Antibiograms were used to profile the phenotypic characteristics of the MDR bacteria based on CLSI guidelines (see Figure S1) [96]. To identify streptomycin-resistant *E. coli*, isolates confirmed to harbor MDR genes were examined for the presence of the Shiga toxin-producing *aadA1* gene, whereas the *blaZ* and *mecA* genes were evaluated to identify penicillin-resistant and methicillin-resistant *S. aureus*.

### 3.6. Determination of the Antibacterial Activity of ChiNPs

The antibacterial properties of ChiNPs against the isolated bacteria were determined using standard disk diffusion methods in accordance with CLSI 2017 guidelines [101]. To this end, a 0.5 McFarland standard of MDR bacteria was prepared and spread onto Muller–Hinton agar plates. Freshly prepared ChiNP-impregnated (100 µL ChiNP solution: 1 mg/disk) blotting paper and commercial antibiotic discs were then placed on the inoculated media, followed by overnight incubation at 37 °C. The antibacterial activity was tested against MDR strains of *S. aureus*, *E. coli*, and a mixed culture (Figure S6). This analysis was conducted in triplicate, and the zone of inhibition was measured using slide calipers. The inhibitory zone data were categorized, and the most effective ChiNP candidate was selected for subsequent MIC analysis.

### 3.7. Determination of Minimum Inhibitory Concentration and Minimum Bactericidal Concentration

The MIC and MBC were determined using the broth dilution method [102] and the drop plate method [103]. To determine the MIC, a stock solution of ChiNPs was homogenized through sonication and serially diluted (2-fold) using PBS in sterile test tubes. Subsequently, 0.5 mL of a previously prepared bacteria suspension (colony-forming unit (CFU): 10^5^/mL; see Figure S3) was added to each test tube containing the diluted ChiNPs, and the tubes were incubated 37 °C for 18 h, followed by inspection of turbidity. The minimum concentration of ChiNPs that inhibited bacterial growth was determined as the MIC. To determine the MBC, inoculums from each tube incubated for 18 h were streaked on selective media and incubated overnight at 37 °C to assess bacterial growth. The lowest concentration of NPs that resulted in bacterial death on the culture plate was identified as the MBC.

### 3.8. Evaluation of the Safety of the Synthesized ChiNPs

To determine the safety of the produced NPs, BHK-21 cells were treated with ChiNPs, and their viability was assessed using the colorimetric MTT assay, as described previously [104]. Briefly, BHK-21 cells were seeded on cell culture dishes at a density of 0.4 × 10^3^ cells and cultured in DMEM supplemented with 1% penicillin and streptomycin and 10% fetal bovine serum (FBS), under conditions of 37 °C temperature, 70% humidity, and 5% CO_2_. Third passage cells were used for the MTT assay. After trypsinization, the cells were seeded on a 96-well plate at a density of 0.5 × 10^3^ cells/well and cultured for 48 h (until reaching 80–90% confluence). They were then treated with the NPs at various doses (MIC) and incubated for an additional 24 h. Next, 10 µL of MTT reagent was added into each well, followed by incubation for 4 h. The MTT-containing medium was discarded, and 100 µL of DMSO was added into each well and reacted for 15 min to dissolve the insoluble formazan dye formed due to MTT reduction by mitochondrial reductase. Finally, the absorbance was measured on a microplate reader using a 450 nm wavelength filter.

### 3.9. Statistical Analysis

Experiments on the inhibitory effects of the chitosan nanoparticle solution were conducted in triplicate, and differences between groups were compared using a one-way ANOVA, followed by a paired *t*-test. Statistical significance levels were determined using the Bonferroni post hoc test, with *p* values below 0.05 indicating statistical significance.

## 4. Conclusions

In this study, we developed an eco-friendly protocol for synthesizing chitosan nanoparticles (ChiNPs) that can be used as an effective alternative to antibiotics. ChiNPs were chosen for this purpose because of their favorable characteristics, including antibacterial activity, biocompatibility, biodegradability, and high ion exchangeability. Our cost-effective ChiNP synthesis protocol utilized a bottom-up approach using various reaction conditions with chitosan powder as the raw material and lemon extract as the crosslinker and stabilizing agent. With this green synthesis method, the highest ChiNP yield was achieved using 20% of the crosslinker for 18 h while stirring at 1100 rpm. The ChiNPs produced under these conditions exhibited particle sizes of 150–250 nm, as revealed by UV–vis, DLS, and XRD analyses. AFM, SEM, and TEM analyses demonstrated that the ChiNPs were spherical in shape. FTIR spectral analysis indicated two distinct stretching, associated with the formation of C–O–C and H_3_N^+^ groups, which can be involved in crosslinking. The ChiNPs exhibited significant antibacterial effects, as reflected by their zones of inhibition against methicillin-resistant (*mecA*) and penicillin-resistant (*blaZ*) *S. aureus*, and streptomycin-resistant (*aadA1*) *E. coli*. Moreover, ChiNPs had a minimal effect on the viability of mammalian cells when used at the MIC dose, indicating that they are biocompatible for in vivo applications.

## Figures and Tables

**Table 1:** Advantages of green synthesis of ChiNPs over chemical synthesis.

Sl No.	Synthesis Protocol	Reducing Agents/Cross-Linker	Compliance with		Antibacterial Activity against MDR Bacteria	Ref.
			Green Synthesis	Biocompatibility		
**1**	Ionic gelation	STPP (Sodium tripolyphosphate)	Do not comply	Do not comply	Not performed	[55]
**2**	Ionic gelation	STPP	Do not comply	Do not comply	Not performed	[56]
**3**	Chemical	NaBH_4_	Do not comply	Do not comply	Not performed	[57]
**4**	Reverse micellar	Sodium bis-(2-ethylhexyl) sulphosuccinate	Do not comply	Do not comply	Not performed	[58]
**5**	Emulsification	Glutaraldehyde	Do not comply	Do not comply	Not performed	[59]
**6**	Nano Precipitation	Acetone, Methanol	Do not comply	Do not comply	Not performed	[58]
**7**	Chemical	NaNO_2_	Do not comply	Do not comply	Not performed	[60]
**8**	Chemical	NaOH	Do not comply	Do not comply	Not performed	[61]
**9**	Ionic gelation	STPP	Do not comply	Do not comply	Not performed	[62]
**10**	Ionic gelation	STPP	Do not comply	Do not comply	Not performed	[63]
**11**	Ionic gelation	STPP	Do not comply	Do not comply	Not performed	[64]
**12**	Ionic gelation	STPP	Do not comply	Do not comply	Not performed	[65]
**13**	Chemical	NaOH	Do not comply	Do not comply	Not performed	[66]
**14**	Green synthesis	Lemon juice	Comply	Comply	Performed	**This work**

**Table 2:** Primer used for characterization of MDR bacteria.

Bacteria	Primers	Sequences (5′-3′)	Targeted Genes	Amplicon Size (bp)	Ref.
*S. aureus*	*nuc*-F	GCGATTGATGGTGATACGGTC	*nuc*	270	[97]
	*nuc*-F	AGCCAAGCCTTGACGAACTAAAC			
	*blaZ-F*	AAGAGATTTGCCTATGCTTC	*blaZ*	377	[98]
	*blaZ-R*	GCTTGACCACTTTTATCAGC			
	*mecA*-F	AACAGGTGAATTATTAGCACTTGTAAG	*mecA*	533	[10]
	*mecA-R*	ATTGCTGTTAATATTTTTTGAGTTGAA			
*E. coli*	*ECO-1*	GACCTCGGTTTAGTTCACAGA	*16sRNA*	585	[99]
	*ECO-2*	CACACGCTGACGCTGACCA			
	*aadA1*F	TATCAGAGGTAGTTGGCGTCAT	*aadA1*	484	[100]
	*aadA1*R	GTTCCATAGCGTTAAGGTTTCATT			

## Data Availability

All the data are provided within this manuscript and Supplementary Materials.

## Supplementary Materials

The following supporting information can be downloaded at: https://www.mdpi.com/article/10.3390/ijms25094746/s1.
