# Supplementary material for: Green Synthesized Chitosan Nanoparticles for Controlling Multidrug-Resistant mecA- and blaZ-Positive Staphylococcus aureus and aadA1-Positive Escherichia coli
Source: Int J Mol Sci. 2024 Apr 26;25(9):4746. doi: 10.3390/ijms25094746 (PMC11083359; doi:10.3390/ijms25094746)
Supplement: Supplementary file 1 [file ijms-25-04746-s001.zip › ijms-2915723-supplementary.pdf]

Supplementary Figures

1. Antibacterial profiling for MDR patterning

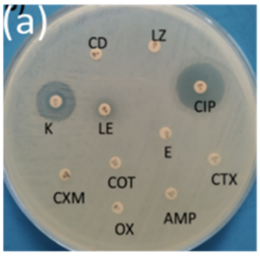

MDR pattern	No. of antibiotic classes	No. of Isolates
COT-CTX-CXM-LZ-LE-E-K-AMP-OX-CD	8	3
COT-CTX-CXM-LZ-E-AMP-OX-CD	7	7
COT-CTX-CXM-E-AMP-OX-CD	6	8

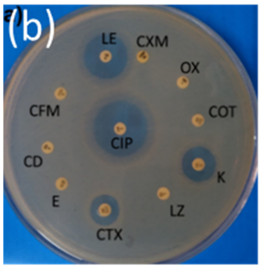

MDR pattern	No. of antibiotic classes	No. of Isolates
COT-CTX-CXM-LZ-LE-E-K-CFM-OX-CD	8	4
COT-CTX-CXM-LZ-E-CFM-OX-CD	7	3
COT-CTX-CXM-E-CFM-OX-CD	6	5
COT-CXM-E-CFM-OX-CD	5	7

Figure S1. Determination of multidrug resistance bacteria following disk diffusion methods using 11 antibiotics against isolated; a) *Staphylococcus aureus* and b) *Escherichia coli*, where COT = Co-Trimoxazole, CTX= Cefotaxime, CXM= Cefixime, LZ= Linezolid, LE= Levofloxacin, E= Erythromycin, K= Kanamycin, AMP= Ampicillin, OX= Oxacillin, CD= Doxycillin, CIP= Ciprofloxacin

2. Determination of suitable conditions for maximum yield of ChiNPs.

For the determination of optimum condition for yielding maximum antibacterial ChiNPs, antibiograms were performed folowing disk diffusion methods, and measured the zone of inhibition. In the case of different ratios of cross-linking agents and chitosan solution, the maximum zone of inhibition of 18.3 mm was revealed from 20% lemon juice cross-linked ChiNPs while 7.5 mm, 11.5 mm, 15.2 mm, and 16.5 mm revealed from 5 to 15% and 25% lemon juice cross-linked ChiNPs, respectively (Figure. S2a). Likewise, in case of different reduction periods, the maximum zone of inhibition 17.5 mm was revealed after 18 h crosslinking periods while 8.5 mm, 7.5 mm, and 14.5 mm were revealed after 6 h, 12 h, and 24 h, respectively (Figure. S2b). Additionally, in case of different stirring speeds, the maximum zone of inhibition 18.8 mm was revealed at 1100 rpm stirring speed while 8 mm, 13.7 mm, and 15.5 mm zone of inhibition were revealed at 700, 900, and 1300 rpm stirring speed, respectively (Figure. S2c). Thus 20% cross-linker for 18 h crosslinking periods at 1100 rpm stirring was considered as an optimum condition for yielding maximum ChiNPs. After confirming the suitable synthesis condition, the ChiNP was subjected to a comparative antibacterial effect analysis of ChiNPs with commercially available antibiotics

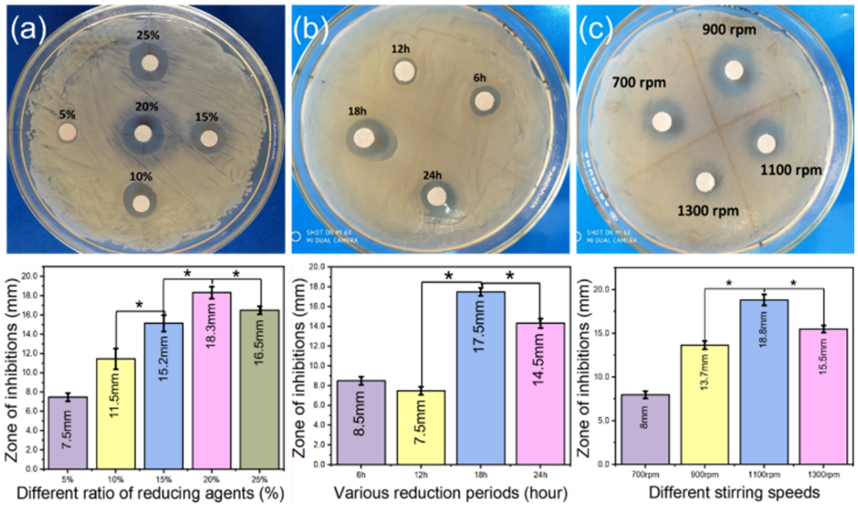

Figure S2. Images showing zones of inhibition of yielded ChiNPs solution synthesized based on (a) concentrations of cross-linker (b) crosslinking periods and (c) stirring speeds.

3. Determination of colony forming unit

The colony-forming unit of MDR bacteria was determined following drop plate methods to obtain the optimum bacteria load before the MIC and MBC test of ChiNPs. The result revealed that the colony-forming unit of Methicillin (*mecA*) resistant *S. aureus* was 9.5x10^5CFU/ml, Penicillin (*blaZ*) resistant *S. aureus* was 1.9x10^8CFU/ml, and Streptomycin (*aadA1*) resistant *E.coli* was 2.1x10^9CFU/ml. After that, the bacterial strains were adjusted to 10^5 CFU/ml by 0.5 McFarland standard to perform the MIC and MBC test.

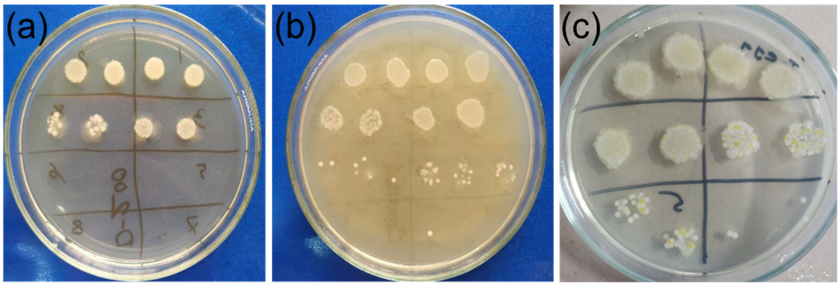

Figure S3. Determination of colony forming unite of a) Methicillin (*mecA*), b) Penicillin (*blaZ*) resistant *S. aureus*, and Streptomycin (*aadA1*) resistant *E.coli*

4. MTT assay

For the determination of cell viability MTT assay was performed using BHK-21 cell line. For this, BHK-21 cells were seeded from the frozen stock of VMH, thawed, and installed in the cell culture plates (25 cm^2) as shown in Figure S4a. The confluent cell culture plate was sub-cultured into two individual plates for the propagation of the cell. After achieving monolayer, cells were spilite and seeded on 96 well plates at a density of 0.4x10^3 cell/well (Figure. S4b) and maintained in a standard cell culture incubator providing all necessary nutrients such as DMEM supplemented with 1 % pens-step and 10 % FBS and incubated aseptically at 37 ^oC with 70 % humidity and 5 % CO_2. After achieving confluent monolayer different doses of synthesized ChiNPs (2×MIC= 30 µl, 1×MIC= 15 µl, 0.5×MIC= 7.5µl, 0.25×MIC= 3.75µl, and 0.125×MIC= 1.875µl as shown in Figure S4c, S4d, S4e, S4f, and S4g) was added into each well without control well (Figure S4h) and incubated for 24 hrs. After incubation 10 µL of the MTT (3-(4,5-Dimethylthiazol-2-yl)-2,5-diphenyltetrazolium bromide) stock solution (0 .5mg/ml) was added to each well. After adding MTT reagent the plate was incubated for 4 hrs for making insoluble formazan. The media of each well were discarded and 100 µL of DMSO into each well and kept for 15 mins for solubilization of formazan measured the OD value was for determining the cell viability through an ELISA reader using a 450nm wavelength filter (Figure S4i).

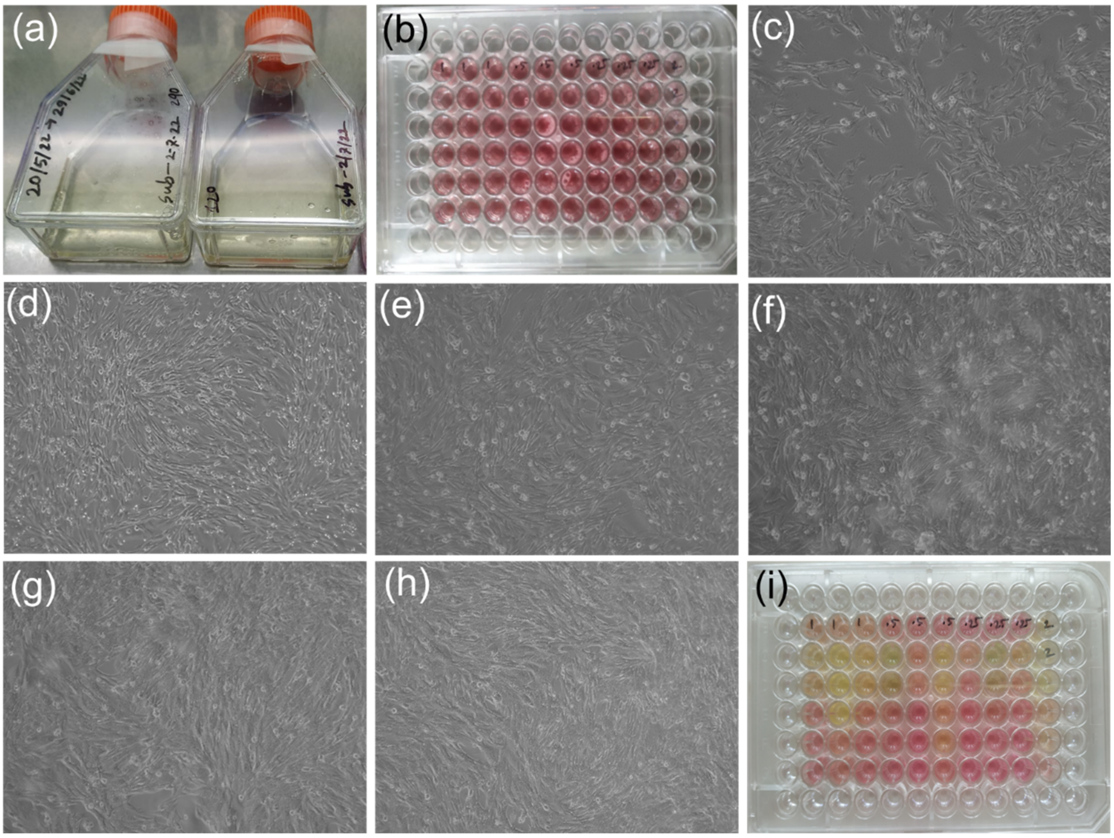

Figure S4. Determination of the cytotoxic effect of ChiNPs using BHK-21 cell; a) cell subculture, b) seeded cell into 96 well plate, c) 2×MIC, d) 1×MIC, e) 0.5×MIC, f) 0.25×MIC, g) 0.125×MIC of ChiNPs treated cell layer, h) control cell, and f) MTT treated plate.

5. Sample collection

A total of 30 shrimp farm water samples were collected from different areas of Satkhria, Bangladesh (Figure. S5). The collected samples were transferred to the laboratory of the Department of Microbiology and Hygiene, Bangladesh Agricultural University, in an icebox maintaining an aseptic condition.

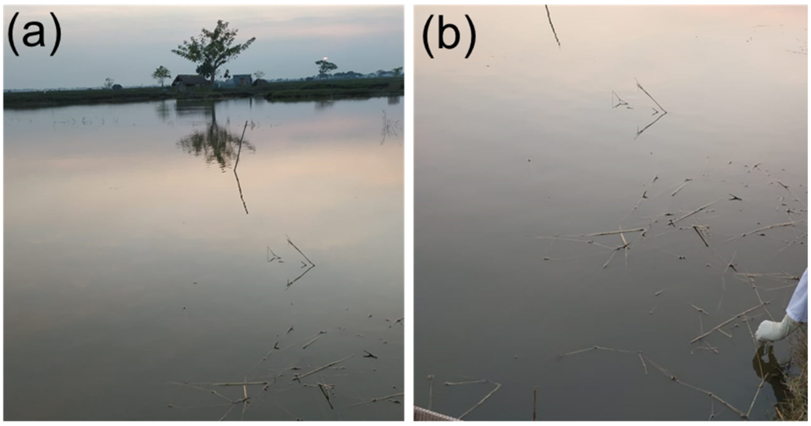

Figure S5. Water sample collection from shrimp farms of Satkhira, Bangladesh**.**

6. Comparative antibacterial activity of ChiNPs

For comparative analysis of antibacterial activity against Gram-positive, Gram-negative and their mixed culture (both G+ and G-), *S. aureus*, *E. coli*, and mixed (for mixed culture equal amounts of 0.5 McFarland *S. aureus* and *E. coli* were mixed and readjust of 0.5 McFarland of the mixed culture before plating) of *S. aureus*, *E. coli* was employed. In the case of Gram-positive *S. aureus* ceftriaxone (CTR), Tetracycline (TE), and ChiNPs showed a 19±9 mm zone of inhibition while 19±9 mm zone of inhibition was found for CTR and ChiNPs against Gram-negative *E. coli.* Additionally, in the case of mixed culture, only ChiNPs showed a 22 mm zone of inhibition.

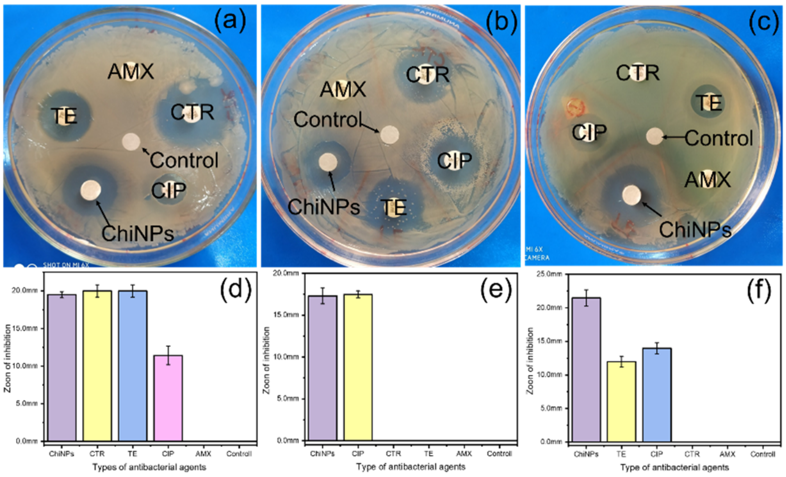

Figure S6. Image showing the comparative antibacterial activity of ChiNPs against a & d) Gram-positive *S. aureus*, b & e) Gram-negative *E. coli*, and c & f) both Gram-positive and Gram-negative (mixed culture).
